# Web-Based Virtual Microscopy of Digitized Blood Slides for Malaria Diagnosis: An Effective Tool for Skills Assessment in Different Countries and Environments

**DOI:** 10.2196/jmir.6027

**Published:** 2016-08-11

**Authors:** Laura Ahmed, Leonard H Seal, Carol Ainley, Barbara De la Salle, Michelle Brereton, Keith Hyde, John Burthem, William Samuel Gilmore

**Affiliations:** ^1^ Manchester Metropolitan University School of Healthcare Science Faculty of Science and Engineering Manchester United Kingdom; ^2^ UK NEQAS Haematology Watford United Kingdom; ^3^ Directorate of Laboratory Medicine Manchester Royal Infirmary Manchester United Kingdom; ^4^ Department of Haematology Manchester Royal Infirmary Central Manchester University Hospitals NHS Foundation Trust Manchester United Kingdom; ^5^ Institute of Cancer Sciences Faculty of Medical and Human Sciences Manchester University Manchester United Kingdom

**Keywords:** Malaria, Virtual microscopy, External quality assessment, Internet

## Abstract

**Background:**

Morphological examination of blood films remains the reference standard for malaria diagnosis. Supporting the skills required to make an accurate morphological diagnosis is therefore essential. However, providing support across different countries and environments is a substantial challenge.

**Objective:**

This paper reports a scheme supplying digital slides of malaria-infected blood within an Internet-based virtual microscope environment to users with different access to training and computing facilities. The feasibility of the approach was established, allowing users to test, record, and compare their own performance with that of other users.

**Methods:**

From Giemsa stained thick and thin blood films, 56 large high-resolution digital slides were prepared, using high-quality image capture and 63x oil-immersion objective lens. The individual images were combined using the photomerge function of Adobe Photoshop and then adjusted to ensure resolution and reproduction of essential diagnostic features. Web delivery employed the Digital Slidebox platform allowing digital microscope viewing facilities and image annotation with data gathering from participants.

**Results:**

Engagement was high with images viewed by 38 participants in five countries in a range of environments and a mean completion rate of 42/56 cases. The rate of parasite detection was 78% and accuracy of species identification was 53%, which was comparable with results of similar studies using glass slides. Data collection allowed users to compare performance with other users over time or for each individual case.

**Conclusions:**

Overall, these results demonstrate that users worldwide can effectively engage with the system in a range of environments, with the potential to enhance personal performance through education, external quality assessment, and personal professional development, especially in regions where educational resources are difficult to access.

## Introduction

The ability to make a successful morphological diagnosis of malaria remains an important and cost-effective health intervention worldwide [1]. However, malaria detection and recognition on blood films require individual skills, knowledge, training, and experience. The effects of misdiagnosis can be very serious [2]. Supporting the quality of diagnosis in different countries and settings can be challenging, particularly for those individuals working in isolation [3,4]. Direct practical instruction and education at the microscope are central to training [5], and glass-slide specimens of malaria-infected blood distributed by reference laboratories provide excellent education in diagnosis [6]. External quality assessment (EQA) has been shown to improve accuracy [7,8]; however, it has also been difficult to implement since the collection and provision of clinical materials are labor intensive, and slide numbers may be limited [9-11].

With facilities for Internet access improving globally, it is now possible to deliver digital slides of blood films to a wide range of geographical locations. Web-based virtual microscope systems [12,13] greatly support this process, and the skillsets have been shown to mirror those used in glass slide diagnosis [14]. The virtual microscope has been used in university education [15-18], EQA schemes [19], and for continuing professional development [20,21].

This study aims to demonstrate the feasibility and effectiveness of the virtual microscope to deliver digital slides of malaria-infected blood with high-quality resolution and to assess how the technique can be used to support skills in malaria diagnosis in developing and developed nations.

## Methods

Participants for this study were recruited with help of the World Health Organization (WHO) and Liverpool School of Tropical Medicine. We used digital slides prepared from 56 cases that had previously been fully validated and distributed as glass slides through the UK National External Quality Assessment Scheme (UK NEQAS) parasitology scheme, confirmed by molecular techniques and consensus opinion of more than 400 laboratories. The cases were selected to represent different slide preparation techniques, stain quality, malaria species, and parasite density. Images were captured using a Zeiss Axio Imager M1 microscope with HRc camera and 63x Plan Apo Chromat 1.4 Oil immersion lens. At least 40 adjacent fields were acquired to create a single large stitched-image. Subsequent image processing used Zeiss software (Axiovision 4.7), then Photoshop CS3 to ensure sharpness and color balance. Diagnostic features were clearly resolved (contrast mask and detail enhancement using Digital Outback Photo add-in), and image size for upload was around 200 megabytes. Images were uploaded to the viewing software (Digital SlideBox, Leica Biosystems) and assessed to ensure the inclusion and accurate rendition of features required for diagnosis. Registered participants were from five countries (ie, Hong Kong, India, Kenya, Lebanon, and Nigeria), and some received financial support for Internet access. In all cases, the participants were asked to suggest their preferred diagnosis (multiple-choice options). Responses were analyzed using Microsoft Excel and GraphPad Prism software (v6.04).

## Results

**Figure 1:**
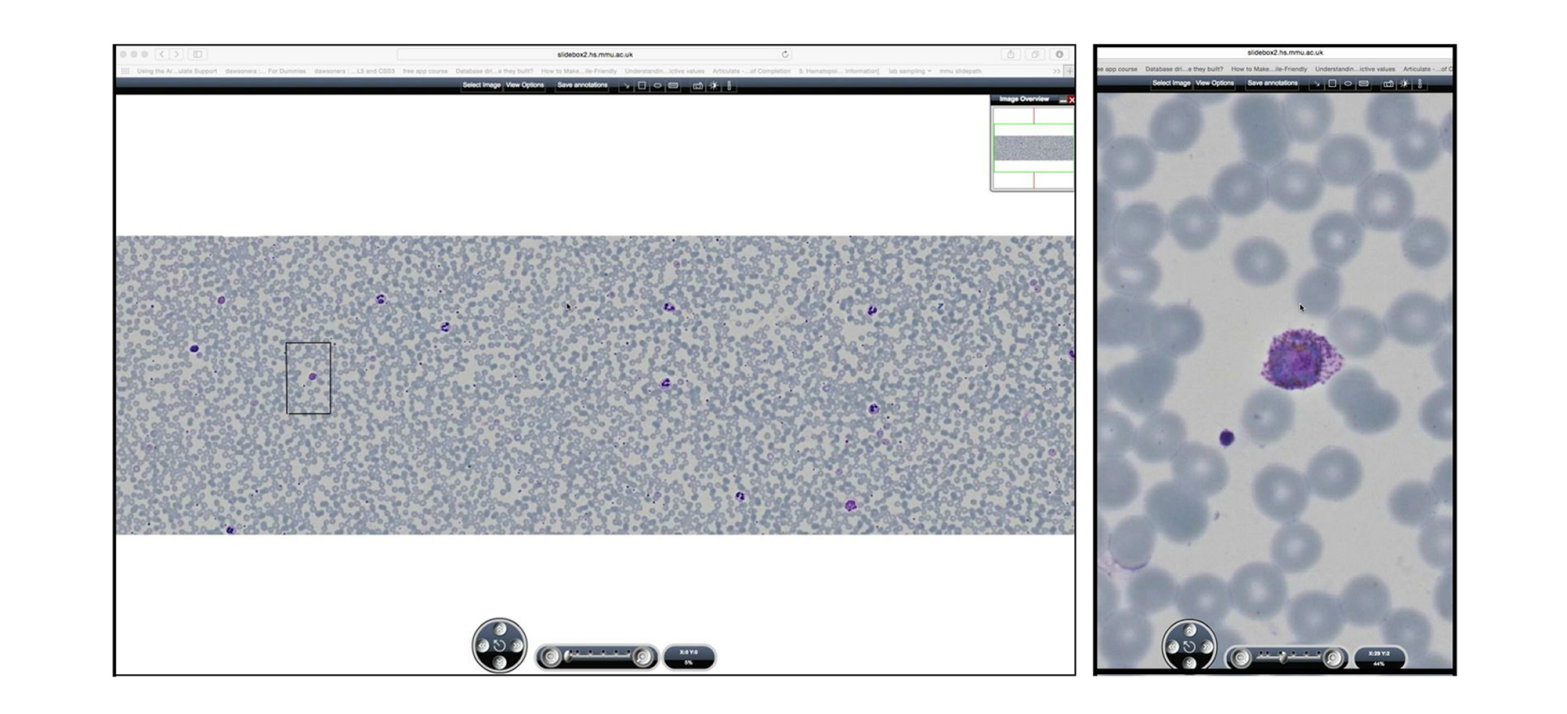
Blood film of parasites shown within Web viewer (Left panel: low power view of whole digital film, showing information buttons and orientation image at top, and navigation and magnification tools at bottom; Right panel: parasite image shown as high power magnification occupying full screen height for the monitor used).

The virtual microscope environment allowed low-power image scanning, navigation, and high-power view assessment (see Figure 1).

**Figure 2:**
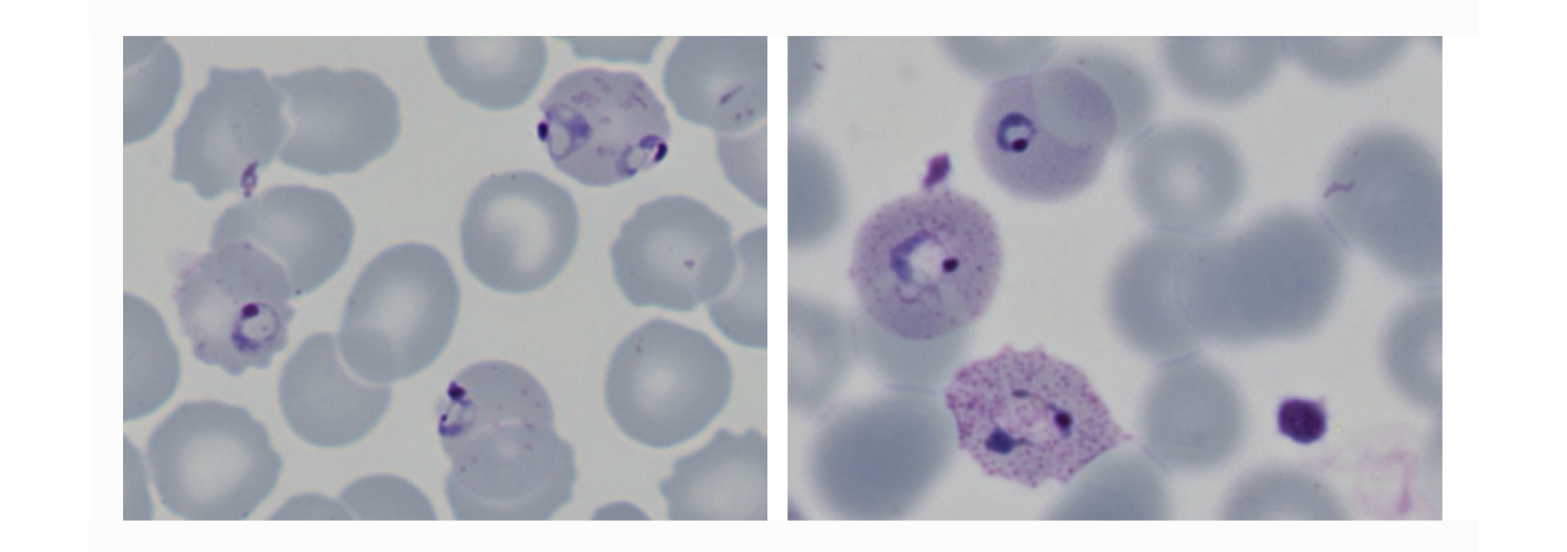
Resolution of features required for parasite detection and species recognition (left panel: digital image of *Plasmodium falciparum* early trophozoites, demonstrating the presence of fine ring together with accole forms with Maurer’s dots and clefts; Right panel: *Plasmodium ovale* parasites with coarse ring forms, James’ (Schűffner’s dots), and cytoplasmic fimbriation of erythrocytes).

**Figure 3:**
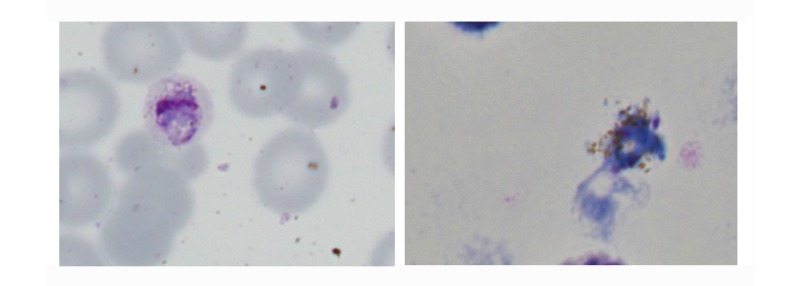
Demonstration of artifact on films presented within Web viewer (Left panel: stain debris visible on a film containing a gametocyte of *Plasmodium malariae*; Right panel: malaria pigment spilling from within a distorted/disrupted parasite [thick film]).

**Figure 4:**
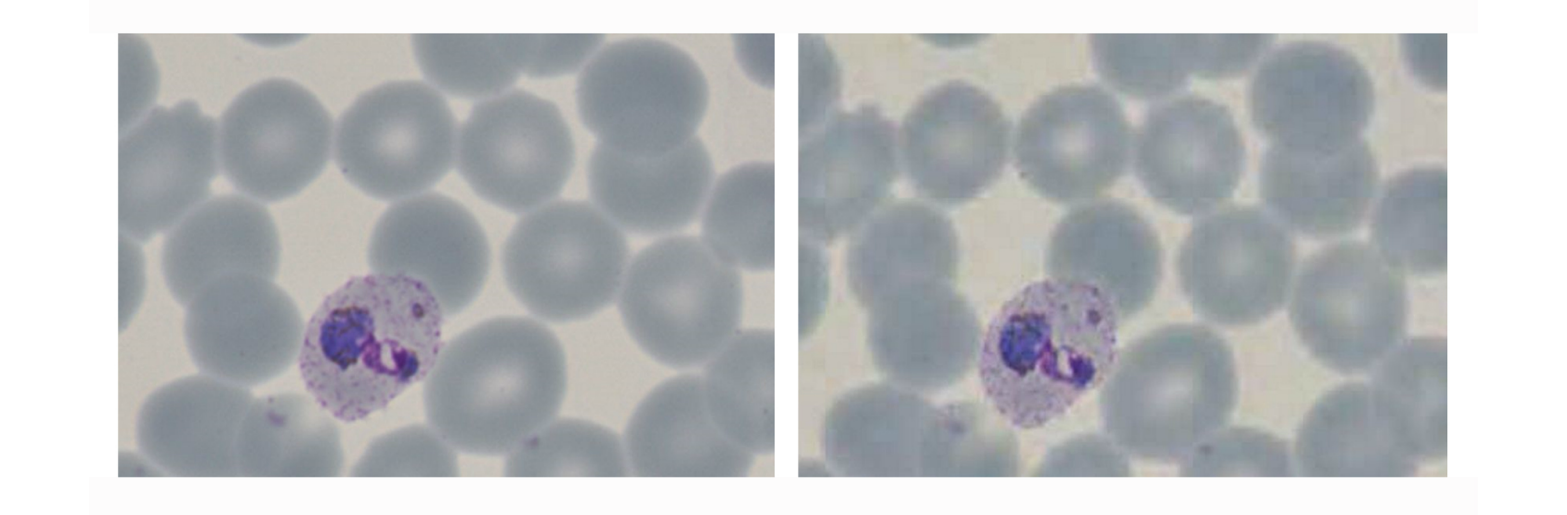
Demonstration of quality considerations applicable to the large digital image: images shown at the time of capture, then subsequently within Web viewer (Left panel: original image quality - resolving parasite, erythrocyte membrane changes, Schűffner’s dots, and malarial pigment; Right panel: screenshot of the same cell following compression and Web delivery showing a subjective difference in reproduction, but retention of all significant diagnostic elements).

The images showed that the initial high-quality image capture clearly resolved those features required for malaria diagnosis, species assignment, or detection of artifact (see Figures 2 and 3). Images were then reassessed following assembly into a large single slide, and finally as a screen capture of a compressed Web-displayed image. Comparison between initial image and Web-browser images revealed an expected subjective loss of definition, but diagnostic detail was retained (see Figure 4).

**Figure 5:**
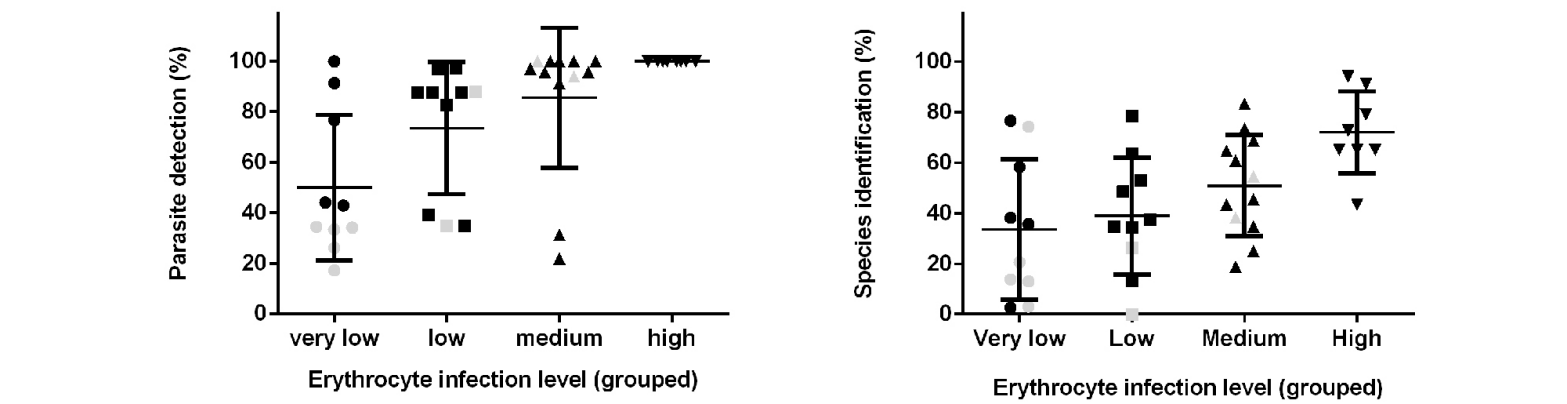
Accuracy of detection and species identification according to number of parasites present on digital blood film. Data groups representing different densities of red cell infection (expressed as parasites per high power field: very low <0.1, low=0.1-0.3, medium=0.3-2, high>2), black=*P. falciparum* cases, gray=non- *P. falciparum*). N= 56 cases, each answered by 22-38 (mean 29) participants. Left panel: parasite detection (mean ±SEM), showing a positive relationship between parasite number and parasite detection (sensitivity=78%) (linear regression analysis of data, r=.48, *P*=.002 for parasite detection); Right panel: species identification (specificity=53%) (r=.54, *P*=.0004).

**Figure 6:**
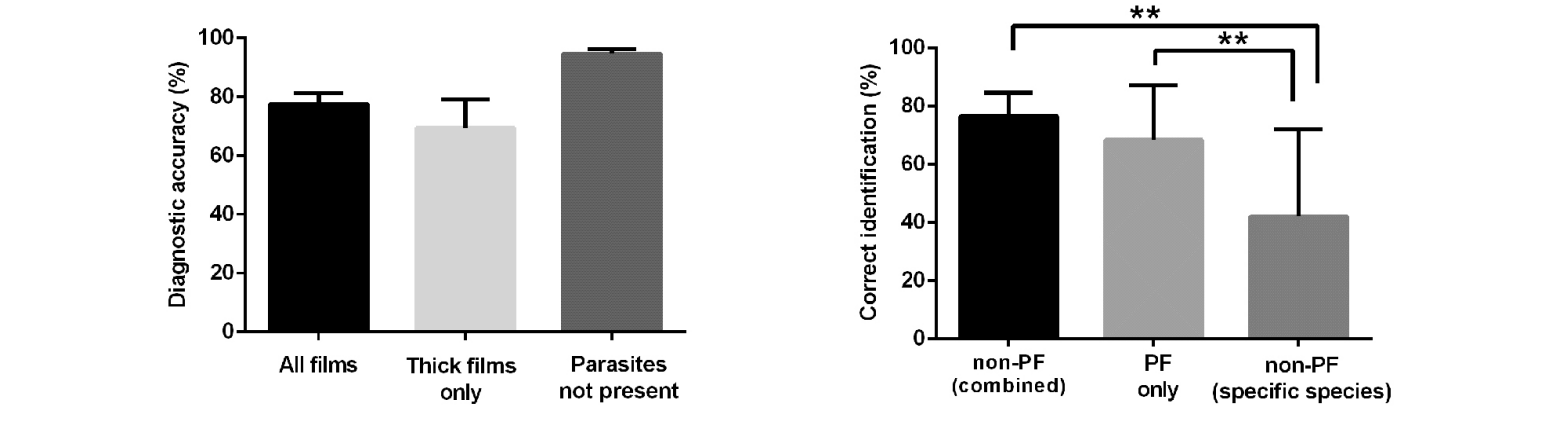
Features determining accuracy of parasite identification by participants using images presented within Web viewer. Left panel: comparison of accuracy of assessment according to type of film or the presence of parasites. Bars represent outcome of detection on thick films or films where no parasites were present compared with the overall responses (bars represent mean ±SEM); n=56 films answered by 22-38 (mean 29) participants. No statistically significant differences were demonstrated. Right panel: comparison of overall parasite detection and detection of non-*P. falciparum* malaria species. Bars represent the mean (±SEM) for each group (*P. falciparum*=31, non-*P. falciparum*=9) analyzed by 38 participants.

The digital slides were then viewed by 38 participants from a range of countries: Hong Kong (n=1), India (n=1), Kenya (n=7), Lebanon (n=6), and Nigeria (n=23). The training and viewing environments differed between groups, and facilities used in different countries were not identical. Although images were viewed predominantly at hospital sites, a significant number used Internet cafés (with financial support). Aspects of the viewing environment were uncontrolled (ie, screen resolution and Internet speed). Although positive experience was reported, the impact of these aspects on results was unclear. Those using Internet cafés had lower rates for sensitivity and specificity for their diagnosis. The mean completion rate was 75% (42/56 cases). Each individual case was completed by a mean of 29 participants (range 22-38). The outcome of slide analysis (indicated by the submitted diagnosis) was compared with parasite density and type. Results indicate that successful recognition and species identification, in keeping with known results of glass slide analysis, were closely linked with the number of malarial parasites present on the slides (see Figure 5). Thick and thin films were assessed with equivalent success, and films containing no parasites were effectively identified. Similarly, in keeping with recognized findings, the recognition that a parasite was *Plasmodium falciparum* or non- *P. falciparum* species was good. However, the precise species-identification for non- *P. falciparum* species was less accurate (see Figure 6).

**Figure 7:**
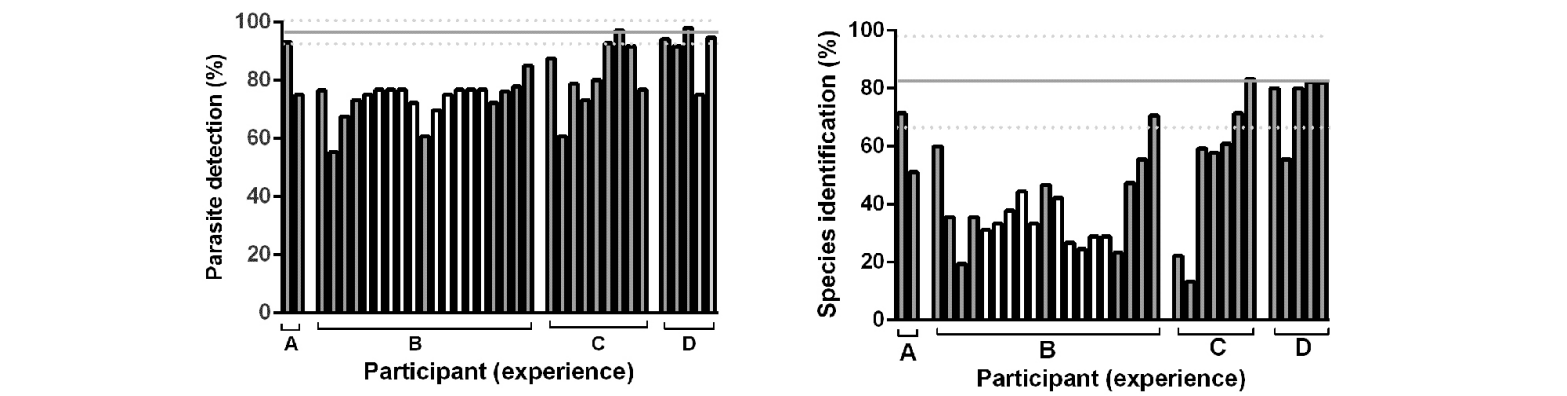
Influence of diagnostic experience performance in parasite identification using consecutive assessments delivered using the image browser. 35 candidates grouped according to length of experience reporting malaria (A: <1yr; B: 1-4yrs; C: 3-9yrs; D: >9yrs). Each bar represents the mean performance for each candidate. The horizontal line represents the performance of UK NEQAS participants using the same case sets on glass slide (solid line=mean, broken line=95% CI, n=435 (mean)). Gray bars represent participants trained at diploma level; all others shown were trained at degree level. Left panel: comparison of individual participants’ detection of malaria parasites; Right panel: comparison of individual participants’ identification of malaria parasites.

**Figure 8:**
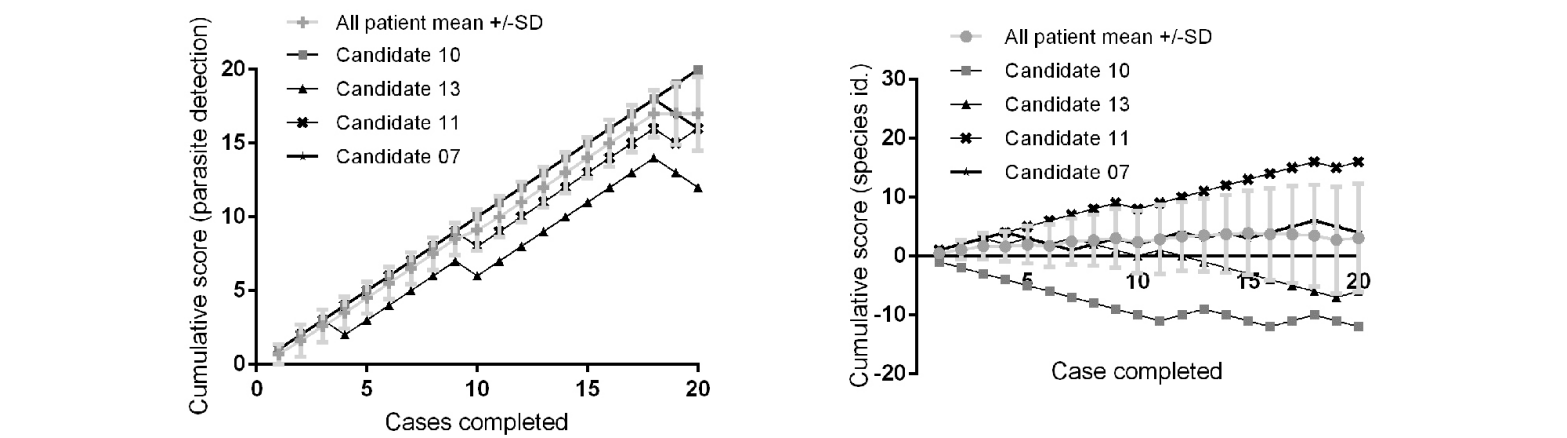
Individual responses compared with overall performance of a cohort over a case series. Left panel (parasite detection): mean cumulative scores ± SD for parasite detection and parasite identification shown for a cohort of 19 participants completing 20 identical cases (correct answer awarded +1 and incorrect is awarded -1). Right panel (parasite identification): representative traces of 4 individual candidates demonstrate tracking of individual performance against the overall mean.

Finally, results were assessed to see whether they could be used to allow participants to assess their individual performance, comparing their results with other participants with similar training or experience (see Figure 7). The performance data across a consecutive case series were also plotted and used to make a cumulative assessment that allowed individuals to compare their performance trend with other users (see Figure 8).

## Discussion

### Principal Findings

Results show that high-quality digital images of malaria-infected erythrocytes prepared as digital microscopy images effectively resolve those morphological features required for diagnosis of malaria and for species recognition. When converted to a Web-compatible format that supports appropriate download speeds, image quality is retained, and cases can be delivered, viewed, and answered by participants in a range of environments in different countries. Overall, the sensitivity for parasite detection and the specificity for species identification was consistent with reports from others using glass slides for comparable cohorts [4,6,22], suggesting that skills applied with the virtual microscope system reflect those made by conventional microscopy. Similarly, those cases that were poorly recognized using the virtual microscope system generally had low parasite number and/or were non- *falciparum* cases—features associated with less accurate diagnosis using glass slides. Finally, those participants achieving highest diagnostic accuracy in this system showed sensitivity of >90% and specificity of around 80%, comparable to results recorded when the same cases were delivered as glass slides to international laboratories as part of established external quality assessment schemes (96% and 85% respectively), confirming that skilled morphologists could achieve high diagnostic accuracy using the system.

On a practical level, the virtual microscope system received positive reviews from participants. They found the system easy to use and access. In this study, the lessons learned were mainly based around communication with remote participants, where contact was only via email. Increased communication correlated with increased participation.

### Conclusion

Web-based delivery allows findings and diagnoses to be rapidly and accurately collected. Analysis of participant responses could be developed to support either individual assessment with longitudinal assessment according to the overall mean, or evaluation of malaria diagnosis skills against a selected peer group. The analysis of performance can be linked to expected performance standards, training level, or personal progression. Linking of this analysis to educational resources presents a real opportunity to support diagnostic skills and to identify sources of error [21], particularly in countries where access to training may be limited. Assessing low parasite density is likely to become more important as many countries move to malaria eradication [23]. It is proposed that the scheme could be adapted and tailored to ensure individual or local relevance (ie, supporting the WHO malaria microscopy quality assurance manual [24]) and can provide opportunities for effective support of malaria diagnosis.

## Abbreviations

EQA: external quality assessment
UK NEQAS: UK National External Quality Assessment Scheme
WHO: World Health Organization
